# A set of novel multiplex Taqman real-time PCRs for the detection of diarrhoeagenic *Escherichia coli *and its use in determining the prevalence of EPEC and EAEC in a university hospital

**DOI:** 10.1186/1476-0711-9-5

**Published:** 2010-01-22

**Authors:** Christoph Hardegen, Sabine Messler, Birgit Henrich, Klaus Pfeffer, Jens Würthner, Colin R MacKenzie

**Affiliations:** 1 Institute of Medical Microbiology and Hospital Hygiene, Heinrich-Heine-University, Universitätsstraße 1, 40225 Düsseldorf, Germany; 2 Translational Pharmacology & Discovery Medicine, GlaxoSmithKline, Gunnels Wood Road, Stevenage SG1 2NY, UK

## Abstract

**Background:**

Accurate measurement of the incidence of diarrhoeagenic *E. coli *in patients with diarrhoea is hindered by the current methods of detection and varies from country to country. In order to improve the diagnosis of diarrhoeagenic *E. coli *(DEC), we developed a set of multiplex TaqMan real-time PCRs designed to detect the respective pathogens from an overnight stool culture.

**Methods:**

Over the period Jan. 2006 to Dec. 2006 all stool specimens (n = 1981) received were investigated for EPEC and EAEC.

**Results:**

Of these, 371 specimens had no growth of *Enterobacteriaceae*. Of the remaining 1610 specimens 144 (8,9%) were positive for EPEC and 78 (4,8%) positive for EAEC. Among the EPEC positive stool specimens 28 (19,4%) were received from the tropical diseases unit, 49 (34%) from the paediatric dept. and 67 (46,5%) from the remainder of the wards. The EAEC were distributed as follows: 39 (50%) - tropical diseases, 19 (24,4%) -paediatrics and 20 (25,6%) other wards. Proportionately more EAEC and EPEC were found in children less than 3 years of age than other age groups. In only 22,2% of the detected EPEC and 23% of EAEC was the investigation requested by hospital staff.

**Conclusions:**

This is, to our knowledge, the first study using a multiplex TaqMan PCR for the successful detection of diarrhoeagenic *E. coli*. In conclusion, due to the high prevalence of DEC detected, investigation of EPEC and EAEC should be recommended as a routine diagnostic test for patients with infectious diarrhoea.

## Background

Infectious diarrhoea is a common complaint among patients seeking medical advice and, despite progress in diagnosis and treatment, remains one of the leading causes of morbidity and mortality worldwide [1,2].

The spectrum of pathogens responsible for such infections varies with age and geographical location. A viral pathogenesis is probably the main cause of diarrhoea in industrialised countries [1,3], however systematic surveys have shown that diarrhoeagenic *E. coli *(DEC) are a common cause of diarrhoea in both developing and developed countries. These pathogens, especially EAEC, may routinely be underestimated as a cause of diarrhoea due to under-representation of requests and difficulty recognising these pathogens in the laboratory [4,5].

To date six categories of DEC have been defined on the basis of specific virulence properties [6,7]. Enterotoxigenic *E. coli *(ETEC) cause diarrhoea via secretion of heat stable (ST) and/or heat labile (LT) enterotoxin. Enteroinvasive *E. coli *(EIEC) strains are closely related to *Shigella *spp. and the responsible genes are carried on the pINV plasmid. Shiga-toxin producing or enterohaemorrhagic *E. coli *(STEC/EHEC) cause both a non-bloody diarrhoea as well as a haemorrhagic colitis which may trigger haemolytic uraemic syndrome (HUS). The virulence properties defining EHEC are the Shiga toxins 1 and 2 (Stx1, Stx2) and, when present, the pathogenicity island LEE ("locus of enterocyte effacement"). Enteropathogenic *E. coli *(EPEC) show localised adherence to the small intestine and cause a histopathological "attaching and effacing (A/E) lesion" mediated through virulence factors encoded within the LEE. Strains additionally containing the EPEC adherence factor (EAF) plasmid have been termed "typical" EPEC, whereas strains that lack the EAF plasmid are referred to as "atypical" EPEC. Enteroaggregative *E. coli *(EAEC or EAggEC) was first described in 1987 [8] and has been associated with acute diarrhoea in children, HIV positive individuals, and as a cause of travellers' diarrhoea [9]. Moreover it has been linked to persistent diarrhoea and even growth retardation in children [10]. Numerous findings suggest that there is an inflammatory process underlying the infection [11,12]. The PCR-target used in this report is a sequence on the plasmid pCVD432 on which the genes coding for the fimbrial structure (aggregative adherence fimbria I) and further virulence factors are located [13]. The sixth category is the diffusely adherent *E. coli *(DAEC) in which the bacteria, unlike EAEC do not adhere to the host cells in microcolonies but rather diffusely over the cell surface. No PCR has been developed yet to detect DAEC strains.

Since DEC cannot be diagnosed adequately by culture and biochemical criteria alone, identification of these strains is difficult. Many PCR techniques detecting various genes coding for virulence traits of the different categories of DEC have been reported [14-18]. We have developed three new multiplex TaqMan PCR assays, designed to be run in parallel, to detect ETEC, EIEC, EHEC, EPEC and EAEC simultaneously.

In addition to many studies from developing countries recent reports underline the importance of EPEC and EAEC in developed countries [5,19-21]. In Germany very limited data are available. Therefore the aim of this study was to evaluate the local importance of EPEC and EAEC in a German University hospital in relation to the requesting clinical department and age group, using the newly developed real-time PCR as a diagnostic tool.

## Methods

### Study population

From January 2006 to December 2006 a total of 1981 faecal samples received for *Salmonella*, *Shigella *and *Campylobacter *spp. culture were included in the screening for EPEC and EAEC. Of these 371 specimens had no growth of *Enterobacteriaceae *hence 1610 samples were tested for EPEC and EAEC by use of the PCR method described below. Stool samples were obtained from most departments of the University Hospital of Düsseldorf, Germany. The patient age ranged from a few days to 98 years.

### DNA preparation

All faecal samples received were plated on MacConkey Agar and incubated for 18 h at 37°C. A bacterial suspension from all colonies was made by rinsing the entire plate with 2 ml of sterile saline. The bacterial suspension was diluted to MacFarland Standard 1,0 (approx. 3*10^8 ^CFU/ml) using distilled water. This suspension was heated at 95°C for 15 minutes. 2,5 μl (corresponding to approximately 7,5*10^5 ^CFU) were applied directly to the multiplex PCR. The remainder of the sample was stored at -20°C.

### TaqMan PCR

Three different multiplex TaqMan PCRs were designed and were all run using the same time and temperature settings on a Biorad iCycler system. A two step amplification profile was used as follows: 95°C for 10 min. followed by 45 cycles of 15 s. at 95°C and 60 s. at 60°C for annealing and elongation. The PCR contained 10 pmol of each primer, 2 pmol of the target probes, 5 pmol of the internal inhibition control probe (all from Eurogentec, Cologne, Germany), 100 copies of inhibition control plasmid, 2,5 μl sample, 12,5 μl of No ROX PCR Mastermix (Qiagen, Hilden, Germany) and was diluted with distilled water to a final volume of 25 μl.

Two target sequences were used for EPEC amplification: a 107 bp sequence of the enteropathogenic *E. coli *adherence factor (EAF) plasmid [22] and a 189 bp long fragment of the intimin gene (*eae*) within the locus of enterocyte effacement based upon an alignment of various published intimin subtype sequences (designed with support of TIB MOLBIOL, Berlin, Germany). The target sequence for EAEC was a 152 bp sequence of the pCVD432 plasmid [23]. The second multiplex PCR was designed to detect ETEC and EIEC. Targets for ETEC were a 107 bp fragment of the ST and a 113 bp sequence of the LT gene. For EIEC a 107 bp sequence of the virulence plasmid essential for invasiveness, pINV was targeted. The third PCR detects EHEC by targeting a 87 bp fragment of the shiga-toxin 1 (*stx*1) and a 82 bp sequence of the shiga-toxin 2 (*stx*2) gene. All probes were labelled with fluorophores and quenchers as listed in Table 1. Isolates of sequenced pathogens served as positive controls while a non pathogenic *E. coli *strain (ATCC 25922) was used as negative control. Since the sequences of pINV and *stx*1 are also present in shigella, in all specimens positive for either of these an infection due to shigella was excluded by culture.

**Table 1:** Primers and probes for multiplex TaqMan PCR

Target	Primer or probe name	Oligonucleotide sequence (5'→3')	**Amplicon size/Accession nr**.
**Multiplex 1:**	**(EPEC/EAEC)**		
EAF	EP-1 for EP-2 rev EP-S probe	GTT CTT GGC GAA CAG GCT TGT C TTA AGC CAG CTA CCA TCC ACC C **Cy5**-AGT ACT GAC GTG CAG GTC GCC TGT TCG-**BHQ-3**	107 bp X76137
			
*eae*	EAE-S for ***EAE-B1 rev*** EAE-B2 rev EAE-TM probe	ACT GGA CTT CTT ATT RCC GTT CTA TG CTA AGC GGG TAT TGT TAC CAG A CCT AAA CGG GTA TTA TCA CCA GA **ROX**-AAT CCT GAT CAA TGA AGA CGT TAT AGC CCA-**BBQ**	189 bp* Z11541/ AB040740
			
pCVD432	EA-1 for ***EA-2 rev*** EA-S probe	AGG TTT GAT ATT GAT GTC CTT GAG GA TCA GCT AAT AAT GTA TAG AAA TCC GCT GTT **FAM**-CAT GTT CCT GAG AGT GCA ATC CCA GAC ATT AC-**TAMRA**	152 bp X81423
			
**Multiplex 2:**	**(ETEC/EIEC)**		
ST gene	ST-1 for ***ST-2 rev*** ST-S probe	CTG GTT TTG ATT CAA ATG TTC GTG TCC TGA GGG AAA GGT GAA AAA GAC **ROX**-TTG ATT TCT TCA TAT TAC CTC CGG ACA TGG CA-**BHQ-2**	107 bp M34916
			
LT gene	LT-1 for LT-2 rev LT-S probe	AGC GGC GCA ACA TTT CAG TTG GTC TCG GTC AGA TAT GTG ATT C **FAM**-TCG AAG TCC CGG GCA GTC AAC ATA TAG A-**TAMRA**	113 bp S60731
			
*ipa*H	Ei-1 for ***Ei-2 rev*** Ei-S probe	GAA CTC AAA TCT TGC ACC ATT CA CGT CCG TCC GAG AAC AAT TAA G **Cy5**-ATC CCC GAC ACC GTT TGT GAG TTT CAC T-**BHQ-3**	107 bp AY206439
			
**Multiplex 3:**	**(EHEC)**		
*stx*1	***slt1-1 for*** slt1-2 rev slt1-S probe	CTT CCA TCT GCC GGA CAC ATA ATT AAT ACT GAA TTG TCA TCA TCA TGC AT **ROX**-AAG GAA ACT CAT CAG ATG CCA TTC TGG CA-**BHQ-2**	87 bp Z36899
			
*stx*2	slt2-1 for ***slt2-2 rev*** slt2-S probe	GAC GTG GAC CTC ACT CTG AAC TG TCC CCA CTC TGA CAC CAT CC **FAM**-TAC TCC GGA AGC ACA TTG CTG ATT CGC-**TAMRA**	82 bp L11079
			
**Internal Control**			
Drosophila simulans +	IC probe	**HEX**-ATG CCT CTT CAC ATT GCT CCA CCT TTC CT-**BHQ1**	AB110070

+The bold, italic labelled primers also detect the internal control plasmid in the respective PCR

*Alignment of various intimin subtype sequences

PCR-based detection methods, especially from faecal samples are prone to inhibition of amplification [24]. As an internal control (IC) specimens were spiked with synthesised nucleotide sequences using an unrelated probe sequence from the retrotransposon Ninja from *Drosophila simulans *(AB110070) in the pCR II-TOPO Vector (Invitrogen, Karlsruhe, Germany) flanked by the respective primers used in the PCR [25]. A serial dilution of targeted template in a MacFarland standard 1,0 apathogenic *E. coli *suspension revealed reliable fluorescence signals to a mean number of 1,5 bacterial genomes per 2,5 μl for all targets. No cross reactivity with various bacteria (*Streptococcus*, *Staphylococcus*, *Enterococcus*, *Pseudomonas*, *Klebsiella*, *Proteus*, *Citrobacter*, *Salmonella*, *Yersinia*, *Campylobacter, Aeromonas caviae, Clostridium difficile *and *Enterobacter *spp.) other than the known positivity with *Shigella dysenteriae *using the EHEC PCR was detected.

## Results

**Figure 1:**
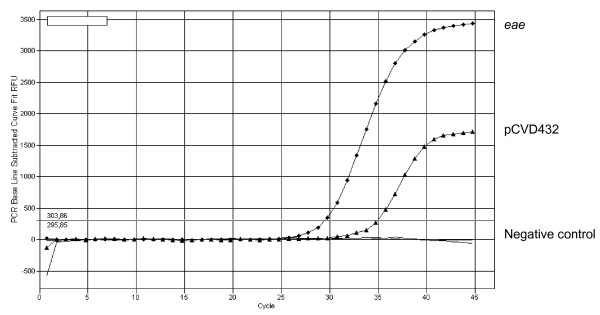
**Amplification plot of a double infection with atypical EPEC and EAEC**.

All stool samples were obtained from the University Hospital of Düsseldorf. Altogether 144 (8,9%) specimens were positive for EPEC and 78 (4,8%) positive for EAEC, including 17 samples that were positive for both pathogens. An example of an amplification plot of a double infection is shown in Fig. 1. From the samples positive for EPEC only 7 (4,9%) were positive for both *eae *and EAF (typical EPEC), making atypical EPEC the predominant pathotype. Five samples were positive for EAF only. All *eae *positive specimens were additionally examined for the presence of *stx*1 and *stx*2 and were all negative. In comparison to these results *Salmonella *spp. was found in 38 (2,4%), *Campylobacter *spp. in 39 (2,4%) and *Shigella *spp. in only 1 (0,06%).

The data were divided into three groups depending on the requesting department: the department for tropical diseases, paediatrics and a last group of all the remaining departments. The department for tropical diseases group consisted of 150 samples only but had a relatively high positivity rate with 28 EPEC positive and 39 EAEC positive samples. 18,7% of all specimens tested within this group were positive for EPEC and 26% for EAEC. Most patients seen in this department have a travel history and, with the exception of only 4 patients, all were between 18 and 65 years of age. From the paediatric wards 458 specimens were tested and 49 EPEC positive and 19 EAEC positive samples were detected showing an prevalence of 10,7% for EPEC and 4,1% for EAEC. From the remaining 1002 samples from all other departments 67 (6,7%) were tested positive for EPEC and 20 (2%) positive for EAEC. Table 2 shows the results.

**Table 2:** Number of stool samples tested according to department and positivity rate for EPEC and EAEC

Department	tested	EPEC (%)	EAEC (%)
tropical diseases	150	28 (18,7)	39 (26,0)
paediatrics	458	49 (10,7)	19 (4,1)
others	1002	67 (6,7)	20 (2,0)

**Figure 2:**
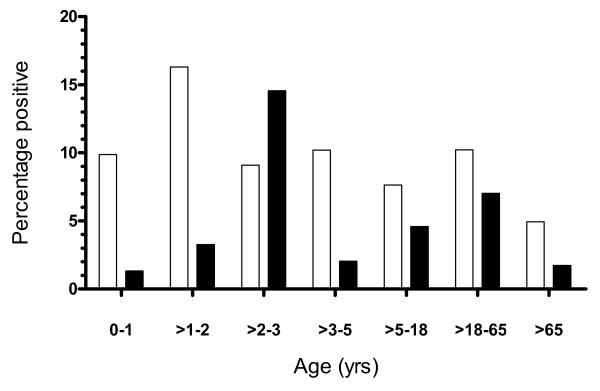
**The percent of specimens positive for EPEC (white square) and EAEC (black square) in age groups compared to all specimens received from patients in the respective age group**.

Since most reports on DEC show different incidences depending on age we divided the samples according to age groups as seen in Fig. 2. A peak prevalence for EPEC was found (16,3%) in the age group between one and two years. In the age groups of patients below one year of age and between two and five years the prevalence varied from 9,1% to 10,2%, which is slightly higher than in the groups between five and 18 years (7,6%) and the group above 65 years of age (4,9%). The prevalence in the group between 18 and 65 years (10,2%) was unexpectedly high, but of the 74 positive samples 26 were sent by the tropical diseases department, indicating a history of travel. With one exception (one positive specimen in the age group below one year), all specimens from the department of tropical diseases were from patients over 18 years of age. For EAEC a clear peak was seen in the age group between two and three years with an prevalence of 14,5%. Below two years of age and between three and five years the prevalence ranged from 1,3% up to 3,3%. None of these samples were from the tropical diseases department. In the age groups between five and 18 and 18 and 65 a smaller peak was found with prevalence of 4,6% and 7% respectively. 1,7% of EAEC positive specimens were from patients over 65. The peak in the group between 18 and 65 years was mostly due to samples from the department for tropical diseases (36 out of 51). In the age group five to 18 years, one specimen and in the group over 65 years, two specimens were received from this department.

In our hospital all stool specimens from children aged up to one year are tested for EPEC routinely, however it was noted that requests for DEC are very rarely made for patients above this age. Therefore we looked at the number of DEC cases found by routine investigations in comparison to the total number of cases found by systematic screening of all specimens received. Table 2 shows the findings for EPEC and EAEC. 15 EPEC positive samples from children below one year of age were detected by routine screening and of the positive results for EPEC or EAEC in the group of samples from children between one and five years of age none was detected by routine request. In the age group between 5 and 18 years of age only one (EPEC positive) specimen was actually requested by the paediatric department and 1 (EAEC positive) specimen from the department for tropical diseases. Between 18 and 65 years a relatively large number of positive results was found in screening all received stool specimens, but only 16 (21,6%) EPEC positive and 15 (29,4%) EAEC positive specimens were detected due to a specific request from the respective department. Furthermore, except for three positive EPEC specimens and one positive EAEC specimen, all of the specimens were sent by the department for tropical diseases. In the group above 65 years in only two of the specimens that were positive for EAEC was the investigation routinely requested, again by the department for tropical diseases.

## Discussion

Detecting DEC relies greatly on PCR techniques and many reports are available on procedures used to identify all categories of DEC [13-18,26]. Most of these techniques however, are based on standard PCR. We have developed a novel multiplex real-time PCR which, with three reactions run parallel to one another, identifies the 5 major categories of DEC simultaneously. To our knowledge this is the first reported use of a TaqMan PCR, which provides both a convenient diagnostic tool and avoids the risk of cross contamination due to post amplification handling.

By rinsing all the colonies from the agar plate with saline rather than picking representative colonies as has been described in the literature, we were able to increase the sensitivity of the PCR. This method is quick, simple and inexpensive. The real-time PCR was able to detect 1,5 genome equivalents per assay.

As reported by the Robert Koch Institute, Berlin, Germany, of all reportable intestinal infections in 2006 in Germany, DEC, excepting EHEC, was the fifth largest group of pathogens after Norovirus, Rotavirus, *Salmonella *and *Campylobacter *spp. In our study reported here, the prevalence of EPEC (8,9%) and EAEC (4,8%) exceeded those for salmonella and campylobacter, each 2,4%. It is possible that the incidence of EPEC and EAEC throughout Germany is far higher than that reported. This discrepancy may very well be due to the low level of routine requests for DEC as we observed in the study period in this hospital. Unfortunately no data can be provided here to compare the incidence of DEC to that of viral pathogens since in this study stool specimens were not systematically tested for viral pathogens. However, in the paediatric population (the age group under 18 years in this study), many specimens were additionally tested for viruses and out of 40 EPEC positive samples additionally tested for viruses, 16 were additionally positive for rotavirus, norovirus or adenovirus. 4 specimens positive for EPEC were additionally positive for *Salmonella *spp. (1) or *Campylobacter *spp. (3). This leaves EPEC as the sole identified pathogen in 50% of all cases. EAEC was the only pathogen detected in 45% of the samples in this age group. In nine cases the specimen was positive for rotavirus, norovirus or adenovirus as well, whereas in three samples *Campylobacter *spp. was found as a co-infection and one specimen was tested positive for *Salmonella *spp. Even though this study provides no systematic data on viral testing we must assume that viral infections outnumber the cases of diarrhoea due to DEC in children. It is possible that more stool samples were submitted for viral detection only and thus the prevalence for EPEC and EAEC found in this study may still be an underestimation in this age group.

A strong association between EPEC and diarrhoea in children has been reported [27], yet we also found a relatively high prevalence for EPEC in older patients.

A large proportion of EPEC (and EAEC) infections may well be associated with travel, since a high number of positive specimens within the group between 18 and 65 years were obtained from the department for tropical diseases. Concerning the association of atypical EPEC with diarrhoea findings are contradictory [27-29], but it continues to be the most prevalent pathotype of EPEC found in industrialised countries [27,29,30], which we also noted in our study. A subset of the heterogeneous group of atypical EPEC has been found in patients with bloody diarrhoea, (in some cases leading to HUS). They resembled EHEC according to their serotype, virulence profile and multilocus sequence types, leading to the assumption that these strains might be EHEC that have at some stage lost the shiga toxin gene during infection [31]. Unusual in this study is the detection of EAF without the *eae *gene as most often *eae *is found alone (atypical EPEC) or with EAF. It is questionable whether strains positive for EAF but not for *eae *should be classified as EPEC and whether they actually are pathogenic. Further investigation is required as to whether this is due to a sequence variation within the intimin gene resulting in a failure of amplification or whether perhaps other bacterial strains have acquired the EAF plasmid.

One limitation of this study is the inability of the method to distinguish between those patients with a double infection with two or more DEC and those patients who are infected with a single bacterium carrying more than one virulence gene. Because we use a pool of bacteria from an overnight culture, it is not possible to trace the result back to a single bacterial clone. In this study we found 17 specimens which were positive for both EPEC and EAEC. In no case was the distinction between a single bacterium carrying both factors or a double infection clinically relevant. Nevertheless, it would be interesting to determine the frequency of multiple gene carriage by a single clone and we are currently prospectively attempting to trace back to the gene-carrying clone(s).

## Conclusions

In our study we find the prevalence for EPEC and EAEC to be unexpectedly high in Düsseldorf, and we conclude that all patients with diarrhoeal disease should be routinely tested for these pathogens, especially children below the age of five, returning travellers and in those specimens in which no other pathogen can be identified. We also demonstrate the use of a novel multiplex PCR for the detection of diarrhoeagenic *E. coli *from stool specimens.

## Competing interests

The authors declare that they have no competing interests.

## Authors' contributions

CH and SM performed all the molecular studies, collected the data and wrote the manuscript, JW and KP designed the study and molecular probes and primers, BH and CM carried out the design and coordination of the study and took part in the writing of the manuscript

All authors have read and approved the final manuscript.

## References

[B1] Cheng AC McDonald JR Thielman NM Infectious diarrhea in developed and developing countries J Clin Gastroenterol 2005 39 757 773 10.1097/01.mcg.0000177231.13770.07 16145337

[B2] Bryce J Boschi-Pinto C Shibuya K Black RE WHO estimates of the causes of death in children Lancet 2005 365 1147 1152 10.1016/S0140-6736(05)71877-8 15794969

[B3] Olesen B Neimann J Bottiger B Ethelberg S Schiellerup P Jensen C Helms M Scheutz F Olsen KE Krogfelt K Etiology of diarrhea in young children in Denmark: a case-control study J Clin Microbiol 2005 43 3636 3641 10.1128/JCM.43.8.3636-3641.2005 16081890 PMC1234006

[B4] Cohen MB Nataro JP Bernstein DI Hawkins J Roberts N Staat MA Prevalence of diarrheagenic *Escherichia coli *in acute childhood enteritis: a prospective controlled study J Pediatr 2005 146 54 61 10.1016/j.jpeds.2004.08.059 15644823

[B5] Nataro JP Mai V Johnson J Blackwelder WC Heimer R Tirrell S Edberg SC Braden CR Glenn MJ Jr Hirshon JM Diarrheagenic *Escherichia coli *infection in Baltimore, Maryland, and New Haven, Connecticut Clin Infect Dis 2006 43 402 407 10.1086/505867 16838226

[B6] Kaper JB Nataro JP Mobley HL Pathogenic *Escherichia coli* Nat Rev Microbiol 2004 2 123 140 10.1038/nrmicro818 15040260

[B7] Nataro JP Kaper JB Diarrheagenic *Escherichia coli* Clin Microbiol Rev 1998 11 142 201 9457432 10.1128/cmr.11.1.142 PMC121379

[B8] Nataro JP Kaper JB Robins Browne R Prado V Vial P Levine MM Patterns of adherence of diarrheagenic *Escherichia coli *to HEp-2 cells Pediatr Infect Dis J 1987 6 829 831 3313248 10.1097/00006454-198709000-00008

[B9] Huang DB Nataro JP DuPont HL Kamat PP Mhatre AD Okhuysen PC Chiang T Enteroaggregative *Escherichia coli *is a cause of acute diarrheal illness: a meta-analysis Clin Infect Dis 2006 43 556 563 10.1086/505869 16886146

[B10] Steiner TS Lima AA Nataro JP Guerrant RL Enteroaggregative *Escherichia coli *produce intestinal inflammation and growth impairment and cause interleukin-8 release from intestinal epithelial cells J Infect Dis 1998 177 88 96 10.1086/513809 9419174

[B11] Bouckenooghe AR DuPont HL Jiang ZD Adachi J Mathewson JJ Verenkar MP Rodrigues S Steffen R Markers of enteric inflammation in enteroaggregative *Escherichia coli *diarrhea in travelers Am J Trop Med Hyg 2000 62 711 713 11304060 10.4269/ajtmh.2000.62.711

[B12] Greenberg DE Jiang ZD Steffen R Verenker MP DuPont HL Markers of inflammation in bacterial diarrhea among travelers, with a focus on enteroaggregative *Escherichia coli *pathogenicity J Infect Dis 2002 185 944 949 10.1086/339617 11920319

[B13] Bischoff C Luthy J Altwegg M Baggi F Rapid detection of diarrheagenic *E. coli *by real-time PCR J Microbiol Methods 2005 61 335 341 10.1016/j.mimet.2004.12.007 15767009

[B14] Aranda KR Fagundes-Neto U Scaletsky IC Evaluation of multiplex PCRs for diagnosis of infection with diarrheagenic *Escherichia coli *and *Shigella *spp J Clin Microbiol 2004 42 5849 5853 10.1128/JCM.42.12.5849-5853.2004 15583323 PMC535216

[B15] Brandal LT Lindstedt BA Aas L Stavnes TL Lassen J Kapperud G Octaplex PCR and fluorescence-based capillary electrophoresis for identification of human diarrheagenic *Escherichia coli *and *Shigella *spp J Microbiol Methods 2007 68 331 341 10.1016/j.mimet.2006.09.013 17079041

[B16] Kimata K Shima T Shimizu M Tanaka D Isobe J Gyobu Y Watahiki M Nagai Y Rapid categorization of pathogenic *Escherichia coli *by multiplex PCR Microbiol Immunol 2005 49 485 492 15965295 10.1111/j.1348-0421.2005.tb03752.x

[B17] Pass MA Odedra R Batt RM Multiplex PCRs for identification of *Escherichia coli *virulence genes J Clin Microbiol 2000 38 2001 2004 10790141 10.1128/jcm.38.5.2001-2004.2000 PMC86652

[B18] Vidal M Kruger E Duran C Lagos R Levine M Prado V Toro C Vidal R Single multiplex PCR assay to identify simultaneously the six categories of diarrheagenic *Escherichia coli *associated with enteric infections J Clin Microbiol 2005 43 5362 5365 10.1128/JCM.43.10.5362-5365.2005 16208019 PMC1248459

[B19] Huppertz HI Rutkowski S Aleksic S Karch H Acute and chronic diarrhoea and abdominal colic associated with enteroaggregative *Escherichia coli *in young children living in western Europe Lancet 1997 349 1660 1662 10.1016/S0140-6736(96)12485-5 9186384

[B20] Kozub-Witkowski E Krause G Frankel G Kramer D Appel B Beutin L Serotypes and virutypes of enteropathogenic and enterohaemorrhagic *Escherichia coli *strains from stool samples of children with diarrhoea in Germany J Appl Microbiol 2008 104 403 410 17887989 10.1111/j.1365-2672.2007.03545.x

[B21] Pabst WL Altwegg M Kind C Mirjanic S Hardegger D Nadal D Prevalence of enteroaggregative *Escherichia coli *among children with and without diarrhea in Switzerland J Clin Microbiol 2003 41 2289 2293 10.1128/JCM.41.6.2289-2293.2003 12791838 PMC156476

[B22] Franke J Franke S Schmidt H Schwarzkopf A Wieler LH Baljer G Beutin L Karch H Nucleotide sequence analysis of enteropathogenic *Escherichia coli *(EPEC) adherence factor probe and development of PCR for rapid detection of EPEC harboring virulence plasmids J Clin Microbiol 1994 32 2460 2463 7814482 10.1128/jcm.32.10.2460-2463.1994 PMC264083

[B23] Schmidt H Knop C Franke S Aleksic S Heesemann J Karch H Development of PCR for screening of enteroaggregative *Escherichia coli* J Clin Microbiol 1995 33 701 705 7751380 10.1128/jcm.33.3.701-705.1995 PMC228017

[B24] Wilde J Eiden J Yolken R Removal of inhibitory substances from human fecal specimens for detection of group A rotaviruses by reverse transcriptase and polymerase chain reactions J Clin Microbiol 1990 28 1300 1307 1696283 10.1128/jcm.28.6.1300-1307.1990 PMC267924

[B25] Rosenstraus M Wang Z Chang SY DeBonville D Spadoro JP An internal control for routine diagnostic PCR: design, properties, and effect on clinical performance J Clin Microbiol 1998 36 191 197 9431945 10.1128/jcm.36.1.191-197.1998 PMC124832

[B26] Persson S Olsen KE Scheutz F Krogfelt KA Gerner-Smidt P A method for fast and simple detection of major diarrhoeagenic *Escherichia coli *in the routine diagnostic laboratory Clin Microbiol Infect 2007 13 516 524 10.1111/j.1469-0691.2007.01692.x 17331124

[B27] Ochoa TJ Barletta F Contreras C Mercado E New insights into the epidemiology of enteropathogenic *Escherichia coli *infection Trans R Soc Trop Med Hyg 2008 102 852 856 10.1016/j.trstmh.2008.03.017 18455741 PMC2575077

[B28] Prere MF Bacrie SC Baron O Fayet O Bacterial aetiology of diarrhoea in young children: high prevalence of enteropathogenic *Escherichia coli *(EPEC) not belonging to the classical EPEC serogroups Pathol Biol (Paris) 2006 54 600 602 17030460 10.1016/j.patbio.2006.07.034

[B29] Trabulsi LR Keller R Tardelli Gomes TA Typical and atypical enteropathogenic *Escherichia coli* Emerg Infect Dis 2002 8 508 513 11996687 10.3201/eid0805.010385 PMC2732489

[B30] Afset JE Bevanger L Romundstad P Bergh K Association of atypical enteropathogenic *Escherichia coli *(EPEC) with prolonged diarrhoea J Med Microbiol 2004 53 1137 1144 10.1099/jmm.0.45719-0 15496393

[B31] Bielaszewska M Middendorf B Kock R Friedrich AW Fruth A Karch H Schmidt MA Mellmann A Shiga toxin-negative attaching and effacing *Escherichia coli*: distinct clinical associations with bacterial phylogeny and virulence traits and inferred in-host pathogen evolution Clin Infect Dis 2008 47 208 217 10.1086/589245 18564929

